# In Vitro Systems for Studying Different Genotypes/Sub-Genotypes of Hepatitis B Virus: Strengths and Limitations

**DOI:** 10.3390/v12030353

**Published:** 2020-03-23

**Authors:** Constance N. Wose Kinge, Nimisha H. Bhoola, Anna Kramvis

**Affiliations:** Hepatitis Virus Diversity Research Unit (HVDRU), Department of Internal Medicine, University of the Witwatersrand, Johannesburg 2000, South Africa; conskinge@yahoo.ca (C.N.W.K.); nimisha124@gmail.com (N.H.B.)

**Keywords:** In vitro systems, transfection, human induced pluripotent stem cells, liver organoids

## Abstract

Hepatitis B virus (HBV) infects the liver resulting in end stage liver disease, cirrhosis, and hepatocellular carcinoma. Despite an effective vaccine, HBV poses a serious health problem globally, accounting for 257 million chronic carriers. Unique features of HBV, including its narrow virus–host range and its hepatocyte tropism, have led to major challenges in the development of suitable in vivo and in vitro model systems to recapitulate the HBV replication cycle and to test various antiviral strategies. Moreover, HBV is classified into at least nine genotypes and 35 sub-genotypes with distinct geographical distributions and prevalence, which have different natural histories of infection, clinical manifestation, and response to current antiviral agents. Here, we review various in vitro systems used to study the molecular biology of the different (sub)genotypes of HBV and their response to antiviral agents, and we discuss their strengths and limitations. Despite the advances made, no system is ideal for pan-genotypic HBV research or drug development and therefore further improvement is required. It is necessary to establish a centralized repository of HBV-related generated materials, which are readily accessible to HBV researchers, with international collaboration toward advancement and development of in vitro model systems for testing new HBV antivirals to ensure their pan-genotypic and/or customized activity.

## 1. Introduction

Hepatitis B is an inflammatory disease of the liver caused by a partially double-stranded enveloped hepatitis B virus (HBV). The virus persistently infects the liver resulting in end stage liver disease, cirrhosis, and hepatocellular carcinoma. Since its discovery in the 1960s [1,2,3,4,5], HBV continues to pose a serious health problem worldwide, accounting for 257 million chronically infected cases in 2015 [6], despite the existence of an effective vaccine. Approved HBV treatment regimens are restricted to interferon and nucleos(t)ide analogues (NAs), but these drugs can only efficiently suppress viral replication, without eliminating the virus [7,8,9]. Interferons, which act as immunomodulators and interfere indirectly with HBV replication, are administered by injection and require long-term use, which can be associated with significant side effects [10]. On the other hand, NAs suppress HBV replication primarily by hindering the process of reverse transcription, a characteristic and crucial step in the HBV replication cycle. A number of the earlier NAs, such as lamivudine, give rise to drug resistance HBV strains, which can result in adverse long-term clinical effects. Therefore, to achieve better treatment outcomes and the ultimate elimination of the virus, there is a need for more potent inhibitors and in vitro systems in which to test them and biomarkers to measure their effect [11]. 

The discovery of the sodium–taurocholate co-transporting polypeptide (NTCP) as the receptor for HBV entry into hepatocytes has stimulated new efforts for the development of novel antiviral strategies and newer systems to test them [12]. HBV is classified into at least nine genotypes and 35 sub-genotypes, with distinct geographical distributions and prevalence [13,14,15]. These (sub)genotypes can have different natural histories of infection, clinical manifestation, and response to antiviral agents [16]. Therefore it is important that the systems used to monitor antiviral efficacy of various agents are able to test the response of the various (sub)genotypes in order to ensure that the antiviral modalities are pan-genotypic (or customized if a pan-genotypic effect is not possible) and can be used in all regions of the world, especially where HBV is endemic, such as in Africa and Asia. Here we review the various in vitro systems that have been used to study the molecular biology of the different (sub)genotypes of HBV and their response to antiviral agents, and we discuss their strengths and limitations.

## 2. Molecular Biology of Hepatitis B Virus 

HBV, the prototype member of the genus *Orthohepadnavirus*, family *Hepadnaviridae*, is the smallest DNA virus infecting humans. It has a partially double stranded 3200 base pair (bp) genome, with four open reading frames encoding seven proteins. The four open reading frames, which are completely or partly overlapping include: The precore/core (preC/C) for HBeAg and HBcAg (capsid protein); P for polymerase (including reverse transcriptase); PreS1/PreS2/S for three envelope proteins (large Hepatitis B surface: LHBs, middle Hepatitis B surface: MHBs, and small Hepatitis B surface: SHBs (HBsAg)); and X for a transcriptional trans-activator protein, x [17].

**Figure 1:**
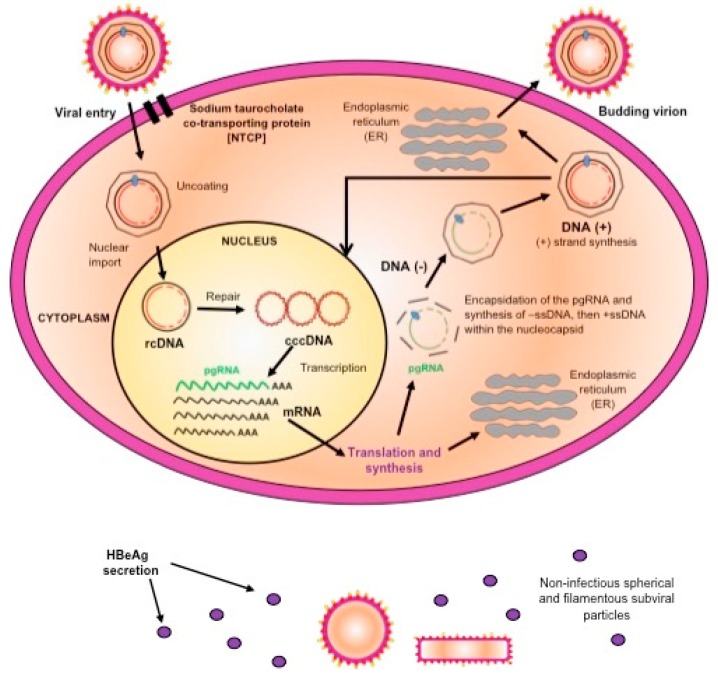
A schematic representation of the replication cycle of hepatitis B virus (HBV).

HBV binds to the NTCP, which is the viral receptor on the hepatocytes [12]. Once in the cytoplasm, the genome is uncoated and imported into the nucleus, where the partially double stranded genome is repaired to give rise to covalently closed circular DNA (cccDNA), which is the template for HBV transcription by the cellular RNA polymerase II [18]. In the cytoplasm, the HBV transcripts are translated into the structural and non-structural proteins. The pre-genomic RNA (pgRNA) together with the viral polymerase are encapsidated into the capsid, where the RNA intermediate is reverse transcribed into the negative strand DNA from which the plus strand is synthesized. The final stage in the viral assembly is the envelopment of the nucleocapsid, with the viral envelope proteins, by budding into the endoplasmic reticulum to form the mature virions, which are then released from the cells. Alternatively, the nucleocapsid can be recycled back into the nucleus, where the relaxed circular DNA is repaired, to maintain the reservoir of cccDNA [18]. In addition to the complete virions, HBV expresses non-infectious filamentous and spherical sub-viral particles composed mainly of HBsAg, the extraparticulate HBeAg, and x protein (Figure 1). 

## 3. Genotypes/Sub-Genotypes of HBV

Sequence heterogeneity is characteristic of HBV because the viral-encoded polymerase, a reverse transriptase, lacks proof-reading ability. HBV has been classified phylogenetically into nine genotypes, A to I [13,14,19,20], based on an intergroup divergence of greater than 7.5% across the complete genome, with a putative 10th genotype, “J”, isolated from a single individual [21], which is a recombinant of genotype C and gibbon HBV [22] and clustering with sub-genotype C4 [23] in the S region. Genotypes A–D, F, H, and I are classified further into at least 35 sub-genotypes, having between a ~4% and 8% intergroup nucleotide difference across the complete genome and good bootstrap support [13]. The genotypes differ in genome length, the size of ORFs and the proteins translated [14], as well as the development of various mutations [24]. HBV has been classified into nine serological subtypes, *ayw*1, *ayw*2, *ayw*3, *ayw*4, *ayr*, *adw*2, *adw*4, *adwq*, *adr,* and *adrq* based on HBsAg heterogeneity [14]. A broad, highly statistically significant relationship exists between serological subtypes and genotypes: *adw* is associated with genotypes A, B, F, G, and H; *adr* with C; and *ayw* with D and E [25], but there are exceptions. The (sub)genotype of HBV can influence the outcome of HBV infection because it can affect the frequency of HBeAg-positivity, the age at which HBeAg loss occurs and the mode of transmission [26]. Therefore, the natural history of HBV infection can differ in different geographical regions [12,13,24,27,28] and references cited therein. Furthermore, the (sub)genotype can affect the response to antiviral therapy [24,27] and possibly vaccination [29,30].

## 4. In Vitro Systems for the Study of HBV

**Figure 2:**
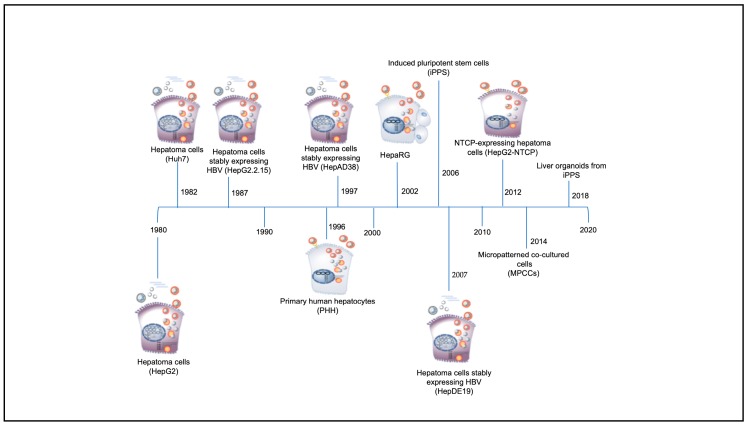
Timeline of discovery of in vitro hepatitis B virus (HBV) systems (adapted from [45]).

Significant features of HBV including its narrow virus–host range and its strong tropism for hepatocytes [31], have led to major challenges in the development of suitable in vivo and in vitro model systems to recapitulate the in vivo human hepatocyte HBV replication cycle [32,33]. The chimpanzee (*Pan troglodytes*) and the macaque (*Macaca fascicularis*) are the only non-human primate models for HBV infection, whereas the tree shrew (*Tupaia belangeri*) is susceptible to HBV infection [33,34]. However, these in vivo animal models have several limitations, including ethical restrictions, high costs and large size in the case of chimpanzees [35], lack of reproducibility in the case of macaques [34,36], and poor infection efficiency and mild and transient infection in the case of the tree shrew [37]. Although a Mauritian macaque colony has been found to be naturally infected with HBV closely related to genotype D [34], this infection could not be recapitulated in vitro or in vivo by others, unless the macaque hepatocytes were transduced by NTCP [36]. A lack of a robust and reproducible in vitro cell culture system that is capable of supporting all the steps of the HBV replication cycle, including infection and formation of cccDNA, has also led to the hindrance of the study of the virus in terms of the mechanisms of the early stages of virus–cell interactions, and the development of anti-HBV drugs [38,39,40,41]. Therefore, to date, most of our understanding on how the HBV functions has come from in vitro and in vivo studies using the duck HBV (DHBV) [42], woodchuck HBV (WHBV) [43], and ground squirrel HBV (GSHBV) [44] models. Over the years, various in vitro cell culture systems have been developed that have enabled the study of the molecular and genetic characteristics of HBV, and the history of their development is depicted in Figure 2.

The various in vitro model systems for the study of HBV have been extensively reviewed elsewhere [32,33,40,41,45,46] and Table 1 provides a brief summary of their strengths and limitations. 

### 4.1. In Vitro Model Systems Based on Hepatoma Cells (HepG2, Huh7, HepG2.2.15, and HepAD38)

Using recombinant HBV DNA constructs carrying over-length HBV genomes, well-differentiated human hepatic cell lines (such as HepG2, Huh6, and Huh7 derived from HCCs) have been transfected to study mechanisms of HBV replication and morphogenesis [59,80,91]. These neoplastic immortal cells are easier to culture and have stable enzyme concentrations when compared to primary hepatocytes. However, they have absent or low expression levels of drug metabolizing enzymes, thus restricting their application [92,93]. Although HBV cannot infect these cell lines most likely as a result of the loss of cell surface receptors during de-differentiation of the hepatocyte [94], they have been widely used and have been invaluable in the study of the various aspects of the life cycle following either: (i) transient transfection or transduction of recombinant HBV DNA using baculoviral, adenoviral, and lentiviral vectors for delivery; or (ii) transfection with recombinant cccDNA (rcccDNA), generated by a minicircle-based technique. This transfection can recapitulate the expression of HBV RNAs and proteins in Huh-7 cells [95] and (iii) the generation of stably transfected cell lines containing integrated HBV DNA genomes [59,91,96]; or (iv) the development of stable rcccDNA-producing cell lines termed HepG2-HBV/loxP [97]. However, these cell culture systems are unsuitable for studying HBV-host cell infection mechanisms such as viral attachment, penetration, and uncoating of the virus and to follow the development of hepatitis and hepatocellular carcinoma (HCC) [98,99]. The rcccDNA systems are excellent for studying the molecular biology of cccDNA and for screening of antiviral agents, which can silence or eliminate HBV cccDNA. Surrogate in vivo mouse models of chronic hepatitis have been established by delivering rcccDNA to mouse hepatocytes using adenoviral vectors [100].

In 1987, the widely used HepG2.2.15 cell line generated by Sell et al. [91], which contains multiple copies of the HBV genotype D genome, was shown to stably express HBV viral gene products. These HepG2.2.15 cells were later used to study the occurrence of spontaneous HBV integrations in the host genome, showing that DNA damage increases the frequency of integration. However, because HBV viral particles produced are generated from chromosomally integrated DNA, the HepAD38 cell line, established by Ladner and colleagues expressing HBV with Tet-OFF and Tet-ON regulatory systems was generated [74]. This was possible following co-transfection of HepG2 cells with plasmids ptetHBV and pUHD15-1neo under the influence of a tetracycline responsive promoter. The successful establishment of this system has permitted for an improved and more strongly controlled platform to study HBV, as well as resulting in a more robust production of viral particles with increased accumulation of cccDNA in the cells.

Another cell line the HepDE19, was generated containing a 1.1 mer HBV transgene mutated in its 5′ pre-core ATG leaving the 3′ pre-core ATG unchanged. Using this strategy, the expression of HBV e-antigen (HBeAg) was now from the episomal DNA and not from the integrated DNA, thus providing a platform for screening cccDNA-targeting drugs on a large-scale [72,101]. A “second-generation” cccDNA reporter cell line, termed HepBHAe82 was developed where an in-frame haemagglutinin (HA) epitope tag was introduced into the precore domain of HBeAg open reading frame in the transgene of HepBHAe82 cells without disrupting any cis-element critical for HBV replication and HBeAg secretion [102]. These developments have allowed for HBV production *in vitro*, however, their use is limited when it comes to studying the regulation of HBV replication. This is because some carry greater-than genome length HBV and a neomycin resistance gene. So, the quest continues to establish stable HBV-expressing cell lines that will enable the study of the relationship between HBV and host genes.

### 4.2. In Vitro Model Systems Based on Primary Human Hepatocytes (PHH)

The discovery of PHHs in 1996 as the only cells to be infected by the authentic HBV in vitro in their fully differentiated form has long enabled in vitro studies of HBV infection in a system with an intact host defense system [53,103]. However, in addition to their limited availability, infection of PHHs with HBV is inefficient because these cells are only viable for a few days after culturing even upon supplementation with dimethyl sulphoxide (DMSO) [54,103]. Also, indefinite maintenance in culture results in loss of liver-specific functions and de-differentiation into fibroblasts, resulting in the impairment of HBV replication, which relies on hepatocyte nuclear factors for transcription within these cells [41,92,94,104]. Further, heterogeneity in the quality of PHHs and the variation in susceptibility to HBV infection results in maintenance difficulty and generation of experiments of poor reproducibility, with great inter-experimental variation that is difficult to control [53,105].

### 4.3. In Vitro Model Systems Based on Differentiated Hepatoma Cell Lines (HepaRG)

In 2002, HepaRG cells, a human hepatoma cell line derived from HCC from a female with chronic hepatitis C infection [106], was shown to be permissive to HBV infection under certain conditions and extended culture time. This cell line contains hepatic progenitor cells that make them susceptible to HBV/HDV infection after differentiation by the addition of DMSO and hydrocortisone [72,73,77,106]. Although HepRG can support HBV infection, cccDNA formation, and secretion of infectious viral particles into the culture medium [72,73], it is an unsuitable system to study the complete HBV life cycle and to evaluate antiviral compounds because of several disadvantages, which include: (i) a complex and time consuming induction of the differentiation process prior to infection with HBV, which requires the addition of DMSO [72]; (ii) cells exhibit heterogeneity in albumin expression and chromosomal abnormalities [41,46]; and (iii) the activity of the number of enzymes involved in drug metabolism varies when compared to PHHs [107,108]. These cells also allow for low to minimal cell-to-cell spread of HBV.

### 4.4. In Vitro Model Systems Based on NTCP Expressing Cell Lines

The middle HBsAg of HBV initially attaches to heparan sulfate proteoglycans (HSP) on the hepatocyte [109]. This is followed by binding of the large HbsAg to the NTCP, which is the essential receptor for HBV infection [12]. NTCP is a sodium-bile acid pump, coded by the SLC10A1 gene, largely expressed in liver cells and is restricted to the sinusoidal plasma membrane. The only established susceptible cell line expressing NTCP is HepaRG. However, its heterogeneity in albumin expression and chromosomal abnormalities do not allow the study the complete HBV life cycle and the evaluation of antiviral compounds. Thus, DMSO-induced differentiation is required [71].

The exogenous expression of NTCP in hepatoma cell lines can render these cell lines susceptible to HBV infection. The establishment of HepG2 and Huh7-based cell lines in which NTCP is over expressed provides a much-needed and easily accessible platform for studying HBV. HepG2-NTCP cells could also be used to identify chemicals targeting key steps of the virus life cycle including HBV cccDNA, and enable the development of novel antivirals against the infection. However, although improved techniques, such as spinoculation, during HBV inoculation and the addition of DMSO in culture media has greatly enhanced infection efficiency of NTCP expressing cells, the system still fails to recap the full HBV life cycle [85]. The reasons being that in contrast to *in vivo*, the system requires very high multiplicity of infection (MOI), the infection is short-lived, does not result in substantial viral spreading, and only a modest amount of cccDNA is detected. As previously reported [103,110,111,112], HBV infection in cell culture systems including adult primary human hepatocytes, HepaRG hepatoma cells, and more recently HepG2-NTCP cells, is enhanced after the addition of polyethylene glycol 8000 (PEG), which promotes the binding of HBV to HSP. Furthermore, a study by Michailidis and coworkers, showed that maintaining PEG in cell culture medium increases infection by at least one order of magnitude, as a result of improved viral spread [113].

### 4.5. In Vitro Model Systems Based on Inducible Pluripotent Stem Cell (iPSCs)

iPSCs are pluripotent reprogrammed cells derived from either adult skin or blood cells and were first discovered by Japanese researchers following the introduction of genes necessary for the expression of a set of transcription factors in specialized adult cells [114]. The iPSCs are capable of self-renewal and differentiation into different body cell types, except for extra-embryonic tissue cells, like the placenta, making them a promising cell source for regenerative therapy in several disease states. To limit donor variability biases and in an attempt to increase hepatocyte availability, in 2006, hepatocyte-like cells (iHeps/HLCs) were differentiated from iPSCs [89,115], and in 2014 Shlomai and colleagues, first established that HBV could infect iPSC-derived HLCs [89]. They showed that HBsAg was efficiently produced in the supernatant after infection; however, the HBsAg levels gradually dropped to background levels. In their study, Sakurai and coworkers observed an increase in HBsAg secretion in culture supernatant up to 17 days following HBV infection in iPS-HLCs [87]. This indicated that iPS-HLCs could support long-term HBV infection. Xia and coworkers supported this finding when they utilized human iPS-derived HLCs as a robust and convenient in vitro model to study HBV [86]. Thus iPS-HLCs provide a promising in vitro HBV infection model and pave the way to dissect the underlying mechanisms of HBV infection and the development of novel anti-HBV drugs.

### 4.6. In Vitro Model Systems Based on Micropatterned Co-Cultured Cells (MPPCs)

Using a combination of microtechnology and tissue engineering techniques, Khetani and Bhatia [116] established a miniature-like multi-well culture system for human liver cells termed micropatterned co-cultured (MPCC) system. The MPCC system was shown to preserve hepatocyte functions for a prolonged period following their plating, thereby serving as a platform for drug toxicity and drug interaction studies. In their study, Shlomai et al. [89] showed that MPCCs support productive HBV infection and, by blocking elements of the hepatocyte innate immune response related to the initiation of IFN-stimulated genes, HBV infectivity can be enhanced. The ability to sustain lengthy and productive HBV infection, makes MPCCs a facile platform for studying virus–host interactions and developing antiviral medications. Despite its advantage in HBV research in providing useful information with respect to the activation of the innate immune response following HBV infection, the MPCC system does not fully support the spread of infection with only minimal infection efficiency of less than 50%. In addition, the system does not provide sustained cccDNA, pre-genomic RNA, HBeAg as well HBsAg production, and re-infection of naïve cells with medium collected from infected cells is not possible, suggesting that the MPCC system is not robust enough to yield highly infectious viral particles [116]. Furthermore, it should be noted that this culture system is time consuming and technically challenging to establish [116,117].

### 4.7. In Vitro Model Systems Based on Liver Organoids

Over the years, the use of human hepatoma cell lines and humanized mouse models in HBV research has increased, but these systems remain poor in recapitulating the complex biology of hepatocytes. Their high cost, difficulty to obtain, and impracticality in drug screening on a large scale has led to the continued search for better cell culture models [118]. Organoid cultures have arisen as a substitute in vitro system to mimic tissues and join the gap between 2D cultures and in vivo mouse/human models. These liver organoids have been established for multiple species derived from induced pluripotent stem cells, embryonic stem cells, hepatoblasts, and adult tissue-derived cells. To some degree, liver organoid cultures recapitulate the complexity and design of the liver and may offer new insights into host-to-organism interactions. Differentiated liver organoids maintain innate immune responses and retain cell polarity of hepatocytes, mimicking the natural entry of HBV, thus permitting their cell-to-cell transmission [119]. Following the establishment of a functional human induced pluripotent stem cell liver organoid (hiPPSC-LO), recent studies show that these are a suitable in vitro culture system to study and model HBV infections [90]. This system is an advancement in the models for generating fundamental knowledge of HBV biology and providing a promising platform toward screening potential new therapies and the development of customized hepatitis treatment.

Although we have come a long way, the development of the ideal in vitro system continues as none of the systems currently available are without limitations. The ultimate in vitro model for the study of HBV infection and its response to various antiviral agents should ideally:

- Express NTCP
- Maintain hepatocyte function and susceptibility to HBV infection indefinitely
- Not require the addition of DMSO to maintain hepatocyte function or PEG to promote infection
- Be capable of being infected with high efficiency with multiple HBV genotypes/sub-genotypes and variants.
- Express the host factors necessary to support HBV infection
- Have high longevity to support the complete viral life cycle
- Have an intact innate immune response
- Have functional pathways
- Be genetically homogeneous
- Recapitulate HBV infection seen in patients or in vivo systems
- Be renewable
- Be of unlimited supply
- Allow for the testing of a wide range of antiviral and immunomodulatory agents
- Be low cost
- Allow for miniaturization
- Be ethically acceptable

## 5. The Use of *In Vitro* Systems to Study Genotypes/Sub-Genotypes of HBV

Although a number of in vitro model systems have been used to study HBV, very few have compared the different (sub)genotypes. Moreover, a panel of strains representative of each (sub)genotype has not been established, making comparisons across studies difficult because of the range of constructs, methods of transfection/infection/transduction, and cell lines used. It is important to note that many earlier studies did not classify the genotype of the virus used [47,53,54,56,57]. Table 2 provides a summary of studies using either viral particles for infection and replication competent HBV DNA, in various delivery vectors, to follow the replication of different serotypes and/or (sub)genotypes in the different in vitro model systems.

As is evident from the above table, the majority of studies to functionally characterize the different (sub)genotypes of HBV in vitro have been carried out on genotype D, with a single study for each of genotypes I [19] and J [130]. The only stably transfected cell line expressing HBV and currently widely available is Hep2.2.15 expressing genotype D and this is the most frequent source of cell-line derived inoculum. It will be important to have cell lines expressing other (sub)genotypes generated and a number of these are currently under development. Although genotype D is a cosmopolitan and diverse genotype with serological subtype *ayw*, it differs from other genotypes by having a 33-nucleotide deletion at the amino terminus of the pre-S1. Therefore, it is important that systems are developed that express HBV without this deletion and with serological subtype *adw* (genotypes A, B, F and H) and *adr* (genotype C) [13].

The genotypes and in some cases sub-genotypes of HBV have distinct geographical distributions [13,14]. In the two regions of the world where HBV is endemic different genotypes prevail: in Asia, genotype B and C; whereas in sub-Saharan Africa, genotypes A, D, and E. Moreover, for the sub-genotype of A, A1 circulating in Africa is different from A2, which is prevailing outside Africa, and they differ in molecular characteristics and natural history [137,138,139,140]. The studies carried out in one region cannot necessarily be extrapolated to other regions. The natural history and response to various antiviral agents can be influenced by the genetic heterogeneity of the (sub)genotypes and therefore this should be taken into account when designing and testing various antiviral modalities. It is important to be aware that even though in some cases the efficacy of antiviral agents may not be affected by sequence heterogeneity, in other cases it is of utmost importance. As all the (sub)genotypes use NTCP to enter to the hepatocyte, the efficacy of entry-inhibitors will not differ between the different (sub)genotypes. On the other hand, sequence heterogeneity of HBV may be challenging when designing guide RNA (gRNA) for the different CRISPR/Cas9 systems and avoiding off-target effects [135]. Thus, in some cases antiviral agents may be pangenotypic, whereas in others the agent will have to be customized for the (sub)genotype prevailing in a particular geographic region and/or population.

## 6. Knowledge Gaps and Future Prospective

Systems to transfect cells with HBV DNA and study its replication have been available for over three decades enabling the study of processes of chronic HBV infection and the identification of many agents that target the later stages of the HBV life cycle. These include HBV DNA transcription, RNA stability, capsid assembly, RNaseH digestion, virion secretion, HBsAg secretion, and reverse transcription inhibitors. Studies of HepG2-NTCP cells and other cell types that express the NTCP transgene have led to the identification of Myrcludex B, an agent that interrupts the entry phase of the HBV life cycle as well as other HBV entry inhibitors. Systems targeting cccDNA could facilitate efforts to find a cure for chronic HBV infection [11]. Despite the advances made, no system is ideal for pan-genotypic HBV research or drug development and therefore their robustness and reliability require further improvement. With the WHO’s vision to end HBV infection by 2030, more work is needed to develop better models that will facilitate the search for a cure for chronic HBV infection. This can be achieved through the establishment of cell culture systems that most strictly resemble human hepatocytes but are more convenient, less costly, limitless in supply or more readily available, and allow more efficient amplification of infection and spread. Improved efforts to stably maintain primary human hepatocyte culture conditions and optimization of iHep cells are of interest. This emphasizes the need to establish a centralized repository of all HBV-related generated materials [141] and protocols [142], which are readily accessible to HBV researchers, with international collaboration toward the advancement and development of in vitro model systems for testing new HBV antivirals with either pan-genotypic or (sub)genotypic activity, depending on the requirement.

## Figures and Tables

**Table 1:** Summary of the in vitro model systems suitable for studying hepatitis B virus (HBV).

Model System		Advantages	Disadvantages	Studies	Reference
**Animal Model Systems**	Primary *Tupaia* hepatocytes (PTH)	Can be infected with HBV	Low HBV infection efficiency; Lack of genetically uniform *Tupaia belangeri* strains High cost	Identification of the sodium taurocholate co-transporting polypeptide (NTCP) as the receptor for HBV infection; Studying cccDNA formation	[12,46,47,48]
	Primary hepatocytes derived from small animal models such as rats and mice	Can support HBV replication; Intact innate immune response	Cannot be infected with HBV and requires bypassing the initial receptor-mediated infection of the cell by direct transfection or transduction of the HBV DNA genome; cccDNA is not formed in mouse cells.	Studying HBV replication from the post-entry stages; Studying the effects of HBV replication and HBV proteins on cellular physiology	[33,49,50,51,52]
**Human Model Systems**	Primary hepatocytes derived from macaques transduced with NTCP	Can support HBV infection	Need to be transduced with NTCP	Macaque primary hepatocytes transduced with NTCP were susceptible to HBV, whereas untransduced cells could not be infected	[36]
	Primary human hepatocytes (PHH)	Ideal and gold standard in vitro model system that can be infected with HBV	Limited availability and lifespan; Loss of hepatocyte function and susceptibility to HBV infection within days of isolation and culture; Unpredictable variability between hepatocyte donors	Studying HBV infection; Studying innate immune response to HBV infection; Studying metabolism and drug toxicity	[32,45,46,49,52,53,54]
	Human fetal hepatocytes	Can be infected with HBV	Limited availability; Limited infection efficiency and apparent absence of viral spreading; Unpredictable variability between hepatocyte donors; Ethical considerations	Studying HBV infection	[55,56]
**Human Model Systems**	Transiently transfected or transduced immortalized tumor-derived or transformed liver cell lines (e.g., Huh7, HepG2)	Can support HBV replication and transcription; Convenient system; Minimal variability; Relatively cheap	Cannot be infected with HBV and requires bypassing the initial receptor-mediated infection of the cell by direct transfection or transduction of the HBV DNA genome; Cellular signaling pathways are significantly altered and therefore do not recapitulate the physiology of normal hepatocytes; Low to minimal cell-to-cell spread; Gene expression in tumor-derived or transformed cell lines differs from that in normal hepatocytes [57]	Molecular characterization of HBV; Studying HBV replication and regulation and comparing replication of (sub)genotypes; Testing efficiency of novel anti-HBV drugs; Drug resistance studies	[33,40,58,59,60,61,62,63,64,65,66,67,68]
	Stably transfected immortalized tumor-derived or transformed liver cell lines, (e.g., HepaRG, HepAD38, HepDE19, HepG2.2.15)	Can support HBV replication and transcription; Good source of virions for infection; HepaRG cell line has morphological and functional features similar to that of PHHs	Cannot be infected with HBV and requires bypassing the initial receptor-mediated infection of the cell by direct transfection or transduction of the HBV DNA genome; HBV expressed from integrated HBV DNA genome and not cccDNA in the case of HepG2.2.15 and AD38 cells; Requires the addition of dimethyl sulfoxide (DMSO) to promote differentiation (HepaRG) and supplementation of PEG to promote viral entry in the case of HepaRG	Molecular characterization of HBV; Studying virus–host interactions Studying HBV replication and regulation; Testing efficiency of novel anti-HBV drugs; Studies addressing the role of the innate immune response in counteracting HBV infection; Testing efficiency of novel anti-HBV drugs	[40,69,70,71,72,73,74,75,76,77,78,79,80,81,82]
	NTCP-expressing hepatoma cell lines	Can be infected with HBV allowing initial stages of infection to be studied; 50% of cells can express HBV versus only 7% in HepaRG; Easy to handle	Requires high multiplicity of infection and addition of PEG for successful infection; Infection is short-lived; Reduced viral spread and cccDNA levels; Majority of studies using cell culture derived HBV have been restricted to genotype D.	Studying the initial stages of HBV infection; Testing antiviral drug efficacy, especially entry inhibitors	[40,52,83,84]
**Human Model Systems**	Hepatocyte-like cells (HLCs/iHeps) derived from pluripotent stem cells (iPSCs)	Reliable source that can be differentiated into mature hepatocytes; Supply unlimited and renewable; Less variable than PHHs; Can be established from different donors with and without HBV infection or liver disease	Expensive system to set up and high degree of expertise is required; Low hepatic function; Unpredictable variability between donors; Certain signaling pathways may be impaired; Inhibition of the innate immune response is required for HBV infection	Studying the host factors essential for HBV infection and replication; Comparison of infection to other in vitro models; Testing of antiviral agents	[12,40,85,86,87,88,89]
	Micropatterned co-cultured cells	Maintains hepatocytic function over weeks after plating; Supports HBV infection; Active innate immune response; Renewable; Minitiarized	Unpredictable variability between hepatocyte donors; Low infection efficiency and apparent absence of viral spreading; Inhibition of the innate immune response is required for infection to occur; HBV viral particles collected from the model system is not infectious; Logistically and technically challenging	Comparison of infection to other in vitro models; Studying drug toxicity and drug interactions; Testing of antiviral agents	[40,89]
	Liver organoids from human induced pluripotent stem cells (iPSCs)	Cells differentiate with strong hepatic function; More susceptible to HBV infection when compared to HLCs derived iPSCs; Prolonged propagation of HBV for up to 20 days; Generation of infectious virions; Recapitulates virus-induced liver dysfunction	Highly sophisticated and labor-intensive system to establish; Some hepatic characteristics may differ from adult hepatocytes	Studying virus–host interactions; Has the potential to be used to study personalized hepatitis treatment	[90]

**Table 2:** Studies conducted for the various genotypes/sub-genotypes of hepatitis B virus (HBV) in different in vitro model systems.

(Sub)Genotype/Serotype of HBV	Model System Based on Different Cell Lines	Source of Viral Particles	Studies	Reference/ Year
Serotype *ayw*	Huh6 Huh7 HepG2	2.1 mer HBV in psV08	Comparison of HBV transfection into different cell lines	[59]/1987
Serotype *adr*	Huh7 Huh2.2 Primary human lens epithelial cells (HLEC1)	2.0 mer HBV in pBR322 1.3 mer HBV in pBR322	Comparison of HBV transfection into different cell lines	[68]/1987
Seroype *ayw*	Huh7	1.0 mer HBV without a vector 1.0 mer HBV in pro-melanin concentrating hormone (pMCH) vector 2.0 mer HBV in pSM2 vector	Functional characterization of HBV	[120]/1995
Sub-genotype D3	HepG2	1.3 mer HBV in a baculovirus vector (Bac-HBV) pBlueBac4.5	Molecular characterization of HBV	[96]/1998
Serotype *ayw*	Primary tupaia hepatocytes (PTH) Huh7 HepG2	1.3 mer in an adenovirus vector (Ad-HBV) pTG9530	Comparison of HBV transduction/infection into different in vitro model systems	[121]/2001
Serotype *ayw*	PTH Rat Chicken Duck Primary human hepatocytes (PHH) HepG2 HEK293	1.3 mer HBV in an adenovirus vector (Ad-HBV) pAdTrack	Comparison of infection efficiency of HBV between different in vitro models	[122]/2001
Genotype A (Serotype *adw*) Genotype D (Serotype *awy*)	HepG2	1.0 mer HBV in pUC19	Regulation of HBV minichromosome	[65]/2006
	Huh7			
Sub-genotype A1, A2, B1, B2 Genotypes C and D	Huh7	1.24 mer HBV in pGEM-T Easy	Functional characterization of HBV genotypes	[123]/2006
Genotype D (Serotype *ayw*)	HepG2	1.1 mer Bac-HBV pTriEx 1.3 mer Bac-HBV pTriEx	Functional characterization of HBV	[124]/2008
Genotype D (Serotype *ayw*)	HepaRG	Supernatant of 1.1 mer Bac-HBV Supernatant of HepG2.2.15	Functional characterization of HBV	[124]/2008
Genotype D (Serotype *ayw3*)	Rat	1.2 mer HBV + 0.1 mer HBx under the control of simian virus 40 early promoter	Studying the effects of HBx on cellular physiology	[125]/2009
Sub-genotype B2	HepG2	1.3 mer HBV in pUC118 vector (Endogenous promoter)	Molecular characterization of HBV mutations	[126]/2009
Genotypes B and D	PTH PHH HEK293 HEK293T Hela HepG2 Huh7 SMCC-7721 BEL-7404	Plasma from a chronic HBV carrier Supernatant of 1.05 mer Ad-HBV and under the control of the cytomegalovirus (CMV) promoter transfected in Huh7 cells (Exogenous promoter)	Identification of NTCP as the receptor for HBV infection	[12]/2012
Sub-genotype A2, Sub-genotype B1 Sub-genotype C2 Sub-genotype D2 Sub-genotype I1	HepG2	1.1 mer Hybrid HBV DNA (1.0 mer sub-genotype A2 HBV isolate + 0.1 mer Serotype *adw2* HBV) in pUC19 vector 1.1 mer Hybrid HBV DNA (1.0 mer sub-genotype B1 + 0.1 mer serotype *adw2* HBV) in pUC19 vector 1.1 mer Hybrid HBV DNA (1.0 mer sub-genotype C2 + 0.1 mer serotype *adw2* HBV) in pUC19 vector 1.1 mer Hybrid HBV DNA (1.0 mer sub-genotype D2 + 0.1 mer serotype *adw2* HBV) in pUC19 vector 1.1 mer Hybrid HBV DNA (1.0 mer sub-genotype I1 + 0.1 mer serotype *adw2* HBV) in pUC19 vector	Testing of drug efficacy for various genotypes of HBV	[127]/2013
	Huh7			
Sub-genotype A1, A2, D3	Huh7	1.28 mer HBV DNA in pCDNA vector with cytomegalovirus (CMV) promoter removed (endogenous promoter)	Molecular characterization of HBV (sub)genotypes	[128]/2014
Genotype A Genotype D	Micropatterned coculture (MPCC) iPSCs-iHeps	Plasma from patients	Comparison of infection efficiencies with different in vitro model systems	[89]/2014
Genotype B	Huh7	1.3 mer HBV DNA in pBluescript KS (+) vector (pHBV1.3B)	Molecular characterization of genotype B	[129]/2015
Sub-genotype A2, B2, C2, D3 Genotype J	Huh7	1.3 mer HBV DNA in pUC57 vector (Endogenous promoter)	Molecular characterization of HBV (sub)genotypes	[130]/2016
	HepG2			
Genotypes A, B, C, D, E, F, G, H	HepG2	1.1 mer in pCDNA-3.1 vector (Exogenous promoter) 1.3 mer in pCDNA-3.1 vector (-CMV) (Endogenous promoter)	Testing of drug efficacy for various genotypes of HBV	[131]/2017
	HepG2-TA2-7			
	HepG2.117			
Genotypes B and C	Huh7	1.1 mer HBV DNA in pCDNA3.1 zeo (−) vector (Exogenous promoter)	Functional characterization of HBV proteins	[132]/2017
Genotypes B, C and D	Huh7 transfected with replication-competent plasmids or cccDNA HepG.2.15 (genotype D) HepAD38 (genotype D)	1.2 mer HBV DNA in pUC19 (pHBV-1.3B, pHBV-1.3C) [128], genotype D and pAAV/HBV1.2 rcccDNA system, including prcccDNA and pCMV-Cre	Examination of *Staphylococcus aureus* clustered regularly interspaced short palindromic repeats (CRISPR)-associated (Cas) system (SaCas9) on HBV replication in transfected and stably transfected cell lines	[133]/2018 *
Sub-genotype A1 (Serotype *adw*2) Sub-genotype D2 (Serotype *ayw*3) Sub-genotype D6 (Serotype *ayw*2) Genotype E (Serotype *ayw*4)	HepG2	1.3 mer HBV DNA in pUC57 (Endogenous promoter)	Molecular characterization of HBV (sub)genotypes	[134]/2019
	Huh7			
Genotype D	HepG2 HepG2-1.5merHBV HeptG2-1.1merHBV	1.1 mer HBV DNA rcccDNA (+/− methylation)	Comparison of anti-HBV activity of 4 orthologous CRISPR/Cas9 systems	[135]/2019 *
Genotypes A and D	iPSC derived HLCs and MPCCs	Infection with three stocks of plasma derived from three different donors. Two stocks were genotype D, the other genotype A	Modelling of HBV-host interactions and anti-HBV drug testing of entecavir and interferon-β (IFN-β)	[89]/2014
Genotypes C and D	iPPSC derived HLCs	Fiber-modified adenovirus (Ad) vector containing genotype C (Ad-HBV: AdK7-gLuc-HBV)	Transduction of iPS-HLCs with HBV and comparison to expression in PHHs and HepG2-NTCP-C4 cells. Testing of antiviral agents entecavir and myrcludex	[87]/2017
Genotypes C and D	iPPSC derived HLCs	Genotype D derived from the culture supernatant of HepG2.2.15.7 cells	Infection of iPS-HLCs with HBV and comparison to expression in PHHs and HepG2-NTCP-C4 cells. Testing of antiviral agents entecavir and myrcludex	[87]/2017
Genotype D	Liver organoids	Infection with genotype D derived from HepG2.2.15	Comparison with infection of iPSC-HLCs, HepG2-TET-NTCP organoids, PHH	[90]/2018

Shaded rows: infection studies with either patient or cell-culture derived inoculum and unshaded rows: transfection or transduction studies. * These are more recent representative studies, used to illustrate the use of various in vitro models to test the CRISPR/Cas9 systems against various genotypes of HBV. The review by Kennedy and colleagues [136] and references cited therein provide a comprehensive overview of the field, which is beyond the scope of the current review.
